# Remarks, Gleanings, and Extracts

**Published:** 1874-05

**Authors:** T. Curtis Smith

**Affiliations:** Ohio


### Remarks, Gleanings and Extracts.

BY T. CURTIS SMITH, M.D., OF OHIO.

*On Neurosal Headache.*—Dr. C. Handfield Jones details ten cases of this disease in the London *Medical Times and Gazette.*

Of these two only were males. This large preponderance on the side of the females, eight to two, is probably accidental, as Dr. Liveing's much larger numbers show the proportion to be about five to four. As to age, four were probably under thirty, and five probably over forty, one about thirty-five. Hereditary tendency to headache was marked in three cases, and may have existed in others where it was not particularly inquired after. Gout was more or less prevalent in the families of cases two, three and ten. Malaria seemed to be the cause in seven. Exhaustion and fatigue acted as provocatives of the attacks in one, four and seven. Visual disorder occurred in one and eight. Hyperæmia of the head was apparent in two, eight and nine. Pallor of the face was marked in seven. As regards the process which conditionates the headache, I am strongly disposed to regard it as consisting essentially in failure of nerve-life, rather than in irregular accumulation and discharge of nerve-force. The state of the sufferers is, for the time, evidently one of prostration; and anything that depresses the general power, either temporarily or permanently, promotes the invasion or rivets the hold of the malady. In case one the patient was evidently over-worked, In case two the attacks were preceded by a sensory paralysis, and their supervention was attended with loss of memory and sense of confusion; moreover, insanity was prevalent in her family. All this points quite in the direction of infirmity or actual failure of nerve-power. Case three was unable to study more than two hours at a time, from brain-fatigue. He had been preparing for examinations. In case four the bad effect of worry and the good effect of recreation were very conspicuous. In case eight nerve-debility was extremely marked, and during the attacks her mental faculties were semi-paralyzed, or rather more. In case nine there was good reason to believe that the sufferer had inherited an infirm nervous system; and there was also the fact, of like import, that she had been laid up for five years with paraplegia, which, as it had disappeared, was probably a neurosis. In case ten the general condition was one of chronic hyperæsthesia and debility. Good reasons exist, as I have long tried to show, why pain should be regarded as a kind of paralysis, and most of the concomitant phenomena of migraine, the anæsthesia, the occasional paralysis, the loss of memory, the aphasia, the confusion of mental faculties, the giddiness, and even the sickness, seem to me to belong to a state of lowered and deteriorated, not exalted or excited, nerve force. Even should there occur in any instance disorder, such as convulsion, which might be deemed indicative of "irregular accumulation and discharge of nerve-force." I should still regard this as essentially homogeneous with the other symptoms, for the reason that it may fairly be referred to loss of the self-controlling power which belongs to nerve-cells in the state of health. One of the marvels of the living body is that most nerve-cells, while duly supplied with blood, possess not only the power of evolving force, but also of regulating the discharge of that force. An athlete may stand ready for some great effort, with his nerve-cells charged, as it were, with force, or that which can produce force, yet not until the word is given is the energy poured out in forceful action. Surely all was in readiness before; the molecular matter of the nerve-cells continuous with the axis cylinders was on the verge of undergoing active change, yet remained quiescent. Of course this occurs under the influence of the will; but the point to be noted is that the will cannot always control a *deteriorated* organ.—*Medical and Surgical Reporter.*

*Ergotin Injections in Prolapsus Ani.*—The eminent surgeon, Von Langenbeck, of Berlin announces that he has lately been treating prolapsus ani "with astonishing success" by hypodermic injections of a solution of ergotin (five to fifteen parts to one hundred of distilled water.) He replaces the bowel, and inserting the point of the syringe about three centimetres in depth in the cellular tissue, throws in from one to two grains of ergotin. This should be repeated every three or four days for three or four weeks, any hard fecal masses in the bowels being first removed by a simple injection. As a means of treating a most obstinate and troublesome complaint, this method, sanctioned by so eminent a name, deserves careful repetition.—*Medical and Surgical Reporter.*

*Weeping in Children.*—M. Trosseau considers it worthy to be considered as an aphorism that an infant is not dangerously ill so long as it sheds tears; and that, on the contrary, absence of weeping indicates a severe disease. He, however, admits that exceptions may occur to this general rule.—*Western Lancet.*

*Animal Vaccination.*—At the stated meeting of the New York Academy of Medicine, held December 4, 1873, Dr. F. B. Foster read a paper on animal vaccination, submitting the following propositions:

I. Vaccination of the human subject with animal vaccination is attended with a degree of success, as regards the protection of vaccination, equal to that obtained by any other method of vaccination.

II. The vaccina produced by animal vaccination is genuine, and is as thoroughly protective against small-pox as the protection obtained by the use of any other form of vaccination.

III. The use of animal vaccine does not entail the dangers of severe local inflammatory affections, or general febrile complications, generally attributed to it by some writers. The dangers or complications are rare. Some unfortunate results have heen reported relative to subsequent erysipelatous affections; but the same results have been realized from the use of other virus, and the objection does not hold against animal vaccine alone.

IV. Animal vaccine, as equal with humanized vaccine, is capable of preservation for a considerable length of time, for transmission to remote parts.

V. The use of animal vaccine eliminates one of the suspected elements of causing syphilis, and helps to silence the anti-vaccine agitations.

VI. Animal vaccine is the most trustworthy safeguard for the supply of material in emergencies.—*N. Y. Medical Journal.*

*A Real Remedy for Onychia Maligna.*—A reviewer in the London *Medical Times and Gazette,* speaking of Prof. Vanzetti's monograph on this disease, says:

"It is a happy thing that a remedy has been discovered which does away with all operative procedures, and cures onychia maligna rapidly and painlessly; namely, *nitrate of lead.* Dr. Moorloose, of Gand, was the first to notify this fact, in 1864, and his paper will be found in the *Abeille Medicale* (No. 16, April 17, 1865), but up to 1868 no one else seems to have made trial of it, nor was it mentioned in any work on materia medica and therapeutics except Trousseau and Pidox's 'Traité de Thérapeutique.' In the eleven cases previously mentioned, which came under Dr. Vanzetti's care in 1868–70, nitrate of lead was used, and with unvarying success. Some of the cases got well with one application of the salt in powder to the ulcerated parts; some required two or three dressings. The ulcers soon lose their foul aspect, and granulate in a healthy manner, and then they rapidly heal. Thus one case of a year's standing was well in two weeks; no other treatment was used. And in all the new nail was healthy and perfectly shaped"—*Medical and Surgical Reporter.*

*Tractile Method of Reducing Strangulated Hernia.*—Dr. Daniel Leasure, of Pittsburgh, Pa., in the *Amer. Jour. of Med. Sci.*, for April, 1864, relates several cases by which the reduction of strangulated hermia was quickly effected after all other tactile efforts had signally failed. Dr. Leasure has practiced this method since 1843, and speaks from personal experience with many cases. The method is to elevate the hips by drawing the limbs over the shoulders of an assistant so that only the head and a little of the shoulders rest on the bed. In other words inverting the erect or standing position of the patient, while the operator presses the contents of the abdomen towards the thorax, and then grasps the hernial tumor as for ordinary taxis, drawing it away from the stricture and if necessary using a little pressure. The doctor states that he has not known this method to fail in effecting reduction, but very properly warns against attempting this method when there is a probability that sphacelation has taken place, as reduction after this occurrence would very likely prove fatal to the patient. He also gives a case where such an accident did occur in the hands of an irregular practitioner.

[We have several times resorted to this method with the most gratifying success, after the taxis had utterly failed in the ordinary positions and methods of reduction. Ed.]

*Pathognomonic Sign of Pertussis.*—M. Bouchut, in the *Revue Medico-Photographique*, states that where pertussis is suspected, if it really exists there will always be found a small ulcer affecting the frenum lingue.—*Clinic.*

---

